# Supplementary material for: Exposure of Cattle Breeding Herds to Naturally Co-Contaminated Zearalenone and Deoxynivalenol: The Relevance of a Urinary Mycotoxin Monitoring System for Herd Health and Food Safety
Source: Toxins (Basel). 2024 Sep 18;16(9):402. doi: 10.3390/toxins16090402 (PMC11436142; doi:10.3390/toxins16090402)
Supplement: Supplementary file 1 [file toxins-16-00402-s001.zip › toxins-3158152-supplementary.pdf]

Supplementary Materials: Exposure of Cattle Breeding Herds to Naturally Co-Contaminated Zearalenone and Deoxynivalenol: The Relevance of a Urinary Mycotoxin Monitoring System for Herd Health and Food Safety

Oky Setyo Widodo, Seiichi Uno, Emiko Kokushi, Osamu Yamato, M. Fariz Fadillah Mardianto, Urara Shinya, Yuto Kano, Chiho Kawashima, Yasuo Fushimi, Tetsushi Ono, Masayasu Taniguchi and Mitsuhiro Takagi

Table S1. Post-hoc comparison analysis of urinary ZEN-DON levels by ELISA and LC-MS/MS in the HF breeding herd

ELISA method

Comparison	ZEN/Cre				DON/Cre			
	Mean dif.	SE	t-value	*P*	Mean dif.	SE	t-value	*P*
Aug-22 - Sep-22	−1204.91	2602.49	−0.46	0.98	−5.15	42.99	−0.12	1.00
Aug-22 - Nov-22	−3793.30	2904.31	−1.31	0.69	235.14	29.46	7.98	0.00
Aug-22 - Mar-23	6363.90	1433.59	4.44	0.01	247.36	29.67	8.34	0.00
Aug-22 - Aug-23	6755.24	1422.27	4.75	0.00	252.59	31.10	8.12	0.00
Sep-22 - Nov-22	−2588.39	3413.09	−0.76	0.94	240.29	32.68	7.35	0.00
Sep-22 - Mar-23	7568.81	2295.51	3.30	0.06	252.51	32.87	7.68	0.00
Sep-22 - Aug-23	7960.15	2288.46	3.48	0.04	257.74	34.17	7.54	0.00
Nov-22 - Mar-23	10157.20	2632.76	3.86	0.03	12.22	10.01	1.22	0.74
Nov-22 - Aug-23	10548.53	2626.61	4.02	0.02	17.45	13.68	1.28	0.71
Mar-23 - Aug-23	391.33	720.61	0.54	0.98	5.23	14.12	0.37	0.99

LC-MS/MS method

Comparison	ZEN/Cre				DON/Cre			
	Mean dif.	SE	t-value	*P*	Mean dif.	SE	t-value	*P*
Aug-22 - Sep-22	0.033	0.819	0.040	1.00	−1.416	0.576	−2.459	0.12
Aug-22 - Nov-22	1.930	0.819	2.356	0.15	1.749	0.576	3.039	0.03
Aug-22 - Mar-23	3.980	0.819	4.859	0.00	1.978	0.576	3.436	0.01
Aug-22 - Aug-23	0.941	0.819	1.149	0.78	1.833	0.576	3.185	0.02
Sep-22 - Nov-22	−1.897	0.840	−2.257	0.18	3.165	0.591	5.359	0.00
Sep-22 - Mar-23	−3.947	0.840	−4.697	0.00	3.394	0.591	5.746	0.00
Sep-22 - Aug-23	−0.908	0.840	−1.081	0.81	3.249	0.591	5.501	0.00
Nov-22 - Mar-23	−2.050	0.840	−2.439	0.12	0.229	0.591	0.387	0.99
Nov-22 - Aug-23	0.989	0.840	1.177	0.76	0.084	0.591	0.142	1.00
Mar-23 - Aug-23	3.039	0.840	3.616	0.01	−0.145	0.591	−0.245	0.99

Cre: creatinine; Mean dif.: mean difference; SE: standard error

Table S2. Results of blood biochemical analyses (mean ± SEM) of the JB and HF breeding herd

JB breeding herd

Parameters	Sampling date					P
	July 2022	August 2022	October 2022	November 2023	December 2023	
GOT[#]	67.25 ± 2.65	54.37 ± 2.62	60.96 ± 3.27	58.3 ± 2.39	57.46 ± 4.49	0.06
GGT	15.88 ± 1.62	14.29 ± 0.98	14.92 ± 1.04	15.98 ± 2.18	18.74 ± 3.33	0.45
FFA*	159.57 ± 15.05	213.83 ± 26.44	275.87 ± 42.98	213.78 ± 40.51	97.78 ± 22.84	0.02
T-Cho*	137.4 ± 11.11	107.48 ± 3.4	97.65 ± 6.36	113.14 ± 5.56	130.8 ± 8.5	0.00
BUN*	11.23 ± 0.41	10.31 ± 0.58	8.58 ± 0.42	8.6 ± 0.45	9.78 ± 0.41	0.00
Glu*	67.36 ± 0.93	58.65 ± 1.46	60.67 ± 2.53	60.5 ± 2.99	62.82 ± 0.72	0.04
Ca[#]	9.38 ± 0.15	9.32 ± 0.13	9.52 ± 0.15	9.06 ± 0.18	8.78 ± 0.28	0.07
IP*	7.7 ± 0.28	6.48 ± 0.24	5.48 ± 0.32	4.84 ± 0.22	5.1 ± 0.13	0.00
Mg	1.98 ± 0.06	2.03 ± 0.07	2 ± 0.04	2.06 ± 0.1	2.08 ± 0.12	0.88
TG*	15.2 ± 1.63	28.37 ± 2.46	21.71 ± 2.32	12.44 ± 2.56	16.56 ± 2.99	0.00
Vit. A*	163.11 ± 6.95	106 ± 3.12	119 ± 4.52	131.4 ± 4.17	122.6 ± 3.31	0.00
Vit. E*	198.22 ± 20.17	180.67 ± 12.15	199.85 ± 22.6	304.6 ± 29.93	326.8 ± 41.88	0.00
3HB*	448.36 ± 27.77	443.26 ± 28.36	330.68 ± 18.63	290.36 ± 34.16	577.4 ± 48.26	0.00
TP*	6.4 ± 0.16	8.04 ± 0.18	7.29 ± 0.23	8 ± 0.3	7.62 ± 0.26	0.00
Alb	3.45 ± 0.06	3.43 ± 0.07	3.42 ± 0.08	3.574 ± 0.06	3.48 ± 0.04	0.72
AG*	1.19 ± 0.05	0.77 ± 0.05	0.94 ± 0.07	0.834 ± 0.08	0.86 ± 0.06	0.00

HF breeding herd

Parameters	Sampling date					P
	August 2022	September 2022	November 2022	March 2023	August 2023	
GOT*	123.30 ± 5.01	106.27 ± 3.61	94.43 ± 4.64	101.06 ± 4.63	111.37 ± 8.88	0.00
GGT*	46.51 ± 3.27	39.21 ± 2.18	32.82 ± 1.73	34.52 ± 3.08	41.67 ± 5.31	0.00
FFA*	190.16 ± 5.65	167.75 ± 14.30	182.89 ± 13.03	230.65 ± 50.89	185.97 ± 20.22	0.03
T-Cho*	208.84 ± 7.66	213.20 ± 6.10	218.52 ± 7.99	249.25 ± 14.69	267.28 ± 15.77	0.00
BUN*	11.49 ± 0.43	15.37 ± 0.56	13.73 ± 0.48	13.29 ± 0.58	9.67 ± 0.55	0.00
Glu*	17.78 ± 1.64	43.57 ± 1.53	67.21 ± 0.82	56.27 ± 1.29	63.56 ± 0.95	0.00
Ca*	9.72 ± 0.08	9.37 ± 0.12	9.17 ± 0.26	9.03 ± 0.19	9.02 ± 0.07	0.00
IP*	7.07 ± 0.12	6.20 ± 0.17	5.98 ± 0.18	6.54 ± 0.13	5.46 ± 0.13	0.00
Mg*	2.50 ± 0.04	2.36 ± 0.04	2.46 ± 0.04	2.55 ± 0.09	2.53 ± 0.04	0.02
TG*	6.92 ± 0.34	6.70 ± 0.38	7.97 ± 0.65	6.34 ± 0.52	12.58 ± 2.14	0.00
Vit. A*	155.60 ± 3.55	197.13 ± 32.65	160.83 ± 5.35	167.78 ± 7.43	130.54 ± 4.06	0.00
Vit. E	577.53 ± 28.49	615.96 ± 26.04	670.83 ± 24.01	633.74 ± 38.39	640.50 ± 32.22	0.24
3HB*	746.58 ± 40.85	773.03 ± 67.71	387.92 ± 22.71	544.06 ± 48.03	691.30 ± 32.67	0.00
TP	7.32 ± 0.07	7.26 ± 0.08	7.30 ± 0.08	7.28 ± 0.10	7.13 ± 0.07	0.39
Alb*	3.84 ± 0.04	3.79 ± 0.04	3.62 ± 0.04	3.87 ± 0.07	3.88 ± 0.04	0.00
AG*	1.13 ± 0.04	1.12 ± 0.03	1.00 ± 0.03	1.17 ± 0.05	1.22 ± 0.04	0.00

*= statistically significant result (P<0.05); [#]= significant tendencies (P = 0.05–0.10)
